# Supplementary material for: Serum, cell-free, HPV-human DNA junction detection and HPV typing for predicting and monitoring cervical cancer recurrence
Source: eLife. 2026 Sep 9;14:RP105741. doi: 10.7554/eLife.105741 (PMC13557711; doi:10.7554/eLife.105741)
Supplement: Supplementary file 2 [file elife-105741-supp1.docx]

**Supplementary Table 1.** PCR primers used.

______________________________________________________________________________________________________________________________________________________

| **Primer ID** | **Forward Primer (5'->3')** | **Reverse Primer (5'->3')** | **Product Size** |
| --- | --- | --- | --- |
| HPV16 E7 long | **CAGCTCAGAGGAGGAGGATG** | **GCCCATTAACAGGTCTTCCA** | 180 bp |
| HPV16 E7 short | **gtgtgactctacgcttcggt** | **tctgagaacagatggggcac** | 110 bp |
| HPV18 E7 | **tgaaattccggttgaccttc** | **ggtcgtctgctgagctttct** | 191 bp |
| HPV35 E7 | **ttgtgtgacagctcagagga** | **acgtatgtcaatgtgtgtgct** | 153 bp |
| HPV39 E7 | **acccgaccatgcagttaatc** | **gctgctgtagttgtcgcaga** | 152 bp |
| HPV45 E7 | **cgagtcagaggaggaaaacg** | **tcctctgccgagctctctac** | 150 bp |
| HPV69 E7 | **gcaacccgaaattgacctac** | **tgttcagcttgtctggcttg** | 114 bp |
| HPV70 E7 | **GGCCGACATTGCAAGAGATT** | **GTTCTTCCCGTCTGGCTAGT** | 162 bp |
| HPV73 E7 | **ccaacaaccgaaattgacct** | **caatggcaaggcatactgtg** | 157 bp |
| **Integration Junction** |  |  |  |
| Patient 1 Junction 1 chr14:99,216,952 – HPV70 L1:6306 | **TTCCAAAAGTGTCTGGCAAA** | **GTGTGTGAATGTGGGGGTGT** | 216 bp |
| Patient 1 Junction 2 chr14:99,216,916 – HPV70 E1:2380 | **TACAGGGACCACCAAACACA** | **CTGGACTGCACACAGACACA** | 168 bp |
| Patient 2 Junction 1 chr11:119,699,940 – HPV16 E1:2248 | **ATGGAGGTGATTGGAAGCAA** | **CTCCAAGGCCTCAGAAGATG** | 159 bp |
| Patient 2 Junction 2 chr11:119,699,962 – HPV16 E2:2907 | **CGCTACAGTGCCTTTTCTCC** | **TCTTTGATACAGCCAGTGTTGG** | 143 bp |
| Patient 3 Junction 1 chrX:137,024,646 – HPV16 E1:934 | **TGACGAGAACGAAAATGACAG** | **AGAGACAGCATGGATGGCTAA** | 114 bp |
| Patient 4 Junction 1 chr8:126,992,703 – HPV18 L1:6887 | **GGGGCATTTAATTTCCACCT** | **AATCCAGCCTGAACCAAAAA** | 173 bp |
| Patient 4 Junction 2 chr8:127,083,002 – HPV18 E1:1708 | **CGTGTACAGATTGGGTTACAGC** | **TGGAAGCCTCATAAAGCAAGA** | 121 bp |
| Patient 5 Junction 1 chr5:1,346,104 – HPV16 L2:5142 | **TTGCTTTACATAGGCCAGCA** | **GTTTGCCAAGAAGCGAGAAC** | 234 bp |
| Patient 5 Junction 2 chr5:1,380,547 – HPV16 L2:5164 | **GTAGGTGAGGCTGCATGTGA** | **TGCTTGAGGAGCCCTTCG** | 227 bp |
| Patient 5 Junction 3 chr5:1,386,461 – HPV16 L1:6256 | **ACTGGAGCGTTTTACCCAAA** | **CCCATGTACCAATGTTGCAG** | 224 bp |
| Patient 5 Junction 4 chr6:106,849,038 – HPV16 L2:4807 | **GTGGCTTCCCAGACACTAGC** | **GTGGCTTCCCAGACACTAGC** | 250 bp |
| Patient 5 Junction 5 chr6:106,849,161 – HPV16 L1:6880 | **ACGACATGGGGAGGAATATG** | **TTCTCTGGGCATGTGTGCTA** | 230 bp |
| Patient 5 Junction 6 chr6:106,847,095 – HPV16 E2:3715 | **GCTCACACAAAGGACGGATT** | **CAAGCTTGCTTTGGAGACAG** | 197 bp |
| Patient 6 Junction 1 chr14:102,789,751 – HPV39 E2:3412 | **ATGGTTTGGGTTTGT** | **TAGTGGCAAATGG** | 137 bp |
| Patient 6 Junction 2 chr14:102,790,038 – HPV39 L1:5934 | **TTATTAGGATCGGGCTGGTC** | **CTGAGTGCAGTGGCTCACAC** | 100 bp |
| Patient 7 Junction 1 chr16:71,705,032 – HPV18 L2:4511 | **GGTGCACCTGATGTAACCAC** | **ATTTCATGGTTGAGCATCTCT** | 150 bp |
| Patient 7 Junction 2 chr16:71,732,359 – HPV18 E1:1857 | **GCTGGAAGAGCCTTGGAAGT** | **CAGTTGCTAAAGGTTTAAGTACGTTG** | 124 bp |
| Patient 8 Junction 1 chr9:87,266,100 – HPV69 E1:1912 | **CAGTACCCACCCAGCACTTT** | **CACACCAGAATGGATAGTAAGACAA** | 121 bp |
| Patient 8 Junction 2 chr9:87,266,133 – HPV69 E2:2171 | **AAACTGTTTTAATACTTGCAAAA** | **CTCCGTAAGCTTGCCCTCT** | 116 bp |
| Patient 9 Junction 1 chrX:13,185,650 – HPV45 L2:4160 | **TTCTGCAAAGAAAGTCAATGG** | **GTGCTGCACGGTGGGATAC** | 202 bp |
| Patient 9 Junction 2 chrX:13,391,674 – HPV45 E2:3539 | **GTCCATGGAAATTGCCAGAT** | **ACGACACGGTATCCGCTACT** | 175 bp |
| Patient 10 Junction 1 chr8:59,436,141 – HPV35 E1: 1974 | **AAGAAGTTTCTTTGGCTGGTAAA** | **TGAAATGGTACAATGGGCATA** | 213 bp |
| Patient 10 Junction 2 chr8:59,436,178 – HPV35 L1: 6996 | **TGCACGCTTGCCTAATCTAA** | **TCAGTATGCTTTGTAAATGTGTCC** | 222 bp |
| Patient 11 Junction 1 chr7:117,590,654 – HPV16 E2: 2338 | **GATTTTTGCAAGGCATACCTAA** | **AGCAATGAGAGAAACTGGTTAAA** | 113 bp |
| Patient 11 Junction 2 chr7:117,594,312 – HPV16 E2: 3270 | **GATCTTGGTCGCTGGATAGC** | **GAAATCTTTGTCTTTTGGCATAGAA** | 104 bp |
| Patient 11 Junction 3 chr7:117,605,110 – HPV16 E2: 3834 | **TGCTACATAGCCAATGGGAAA** | **CGTGACCAATTTTTGTCTCAA** | 121 bp |
| Patient 12 Junction 1 chr2:63,048,331 – HPV16 E7: 787 | **ATGGGCTGAGCGTTAAGAAA** | **CTGCAGGATCAGCCATGGTA** | 127 bp |
| Patient 12 Junction 2 chr2:63,048,344 – HPV16 E1: 2103 | **AAAGGATTGTGCAACAATGTG** | **GGGGAAGTTGGGAGGTATCA** | 110 bp |
| Patient 13 Junction 1 chr6:134,482,206 – HPV18 E1: 2297 | **GTGCAATTTTTGCGATACCA** | **GCTGGCGTGTCCTCTAACTC** | 183 bp |
| Patient 13 Junction 2 chr6:134,527,589 – HPV18 L1: 6444 | **TCGTGGCTCAATTTTGTAAATG** | **GTGAAGCACGCATACCTGTG** | 153 bp |
| Patient 14 Junction 1 chr15:89,108,581 – HPV18 E2: 3211 | **CATGCGAGGAACTATGGAAT** | **CACAACTTACAGTGCGGAAA** | 150 bp |
| Patient 14 Junction 2 chr15:89,118,708 – HPV18 E2: 3946 | **CCAGCATTGGTTCCTGAGTA** | **GGAACAAAATTTGGTGGGATA** | 109 bp |
| Patient 14 Junction 3 chr15:89,118,716 – HPV18 E2: 4003 | **GGCACGTTCGTATCACCATA** | **TGCCACAGTCAGCTTTCTTTT** | 116 bp |
| Patient 14 Junction 4 chr15:89,144,328 – HPV18 E1: 1201 | **AAATCATCTTGATTGAGGCTAA** | **CCAAAAATATACCAAGGCTTGAC** | 150 bp |
| Patient 15 Junction 1 chr4:67,867,259 – HPV73 E1: 2652 | **TCCTGACACGCACTTAAACG** | **TGGTGAGAGATAGAGGTCCAGTT** | 197 bp |
| Patient 15 Junction 2 chr4:68,002,635 – HPV73 E1: 2488 | **GGACCAAATGAAAGATTACAGGA** | **CACCTGGATGCTGGACGTA** | 145 bp |
| Patient 16 Junction 1 chrX:35,045,996 – HPV16 E2: 3968 | **AACGCAGAGGCTGCTGTTAT** | **TCACTTTAATCAAGGCTGTCTGA** | 101 bp |
| Patient 16 Junction 2 chrX:35,046,002 – HPV16 E2: 3077 | **CCTTGGAGGTTACTGAATACCG** | **TGGACATTACAAGACGTTAGCC** | 122 bp |
