# Supplementary material for: Serum, cell-free, HPV-human DNA junction detection and HPV typing for predicting and monitoring cervical cancer recurrence
Source: eLife. 2026 Sep 9;14:RP105741. doi: 10.7554/eLife.105741 (PMC13557711; doi:10.7554/eLife.105741)
Supplement: Supplementary file 3 [file elife-105741-supp2.docx]

**Supplementary Table 2.** Patient clinical parameters

____________________________________________________________________________________________________

**Variable	Cohort (n=16)	No Recurrence (n=11)	Recurrence (n=5)	p-value^a^**

**Age (years)**	57.1	52.6 (12.4)	67 (12.0)	0.048

**Histology**	0.25

Squamous Cell	12 (75.0)	7 (63.6)	5 (100.0)

Adenocarcinoma	4 (25.0)	4 (36.4)	0 (0.0)

**Stage**	0.28

Early (1A-1B2)	8 (50.0)	7 (63.6)	1 (20.0)

Late (1B3-IVA)	8 (50.0)	4 (36.4)	4 (80.0)

**HPV by Clade**	0.013

Clade 7	6 (37.5)	2 (18.2)	4 (80.0)

Clade 9	8 (50.0)	8 (72.7)	0 (0.0)

Other	2 (12.5)	1 (9.1)	1 (20.0)

**Baseline cfDNA** (ng/ml)	49.1 [21.4,336]	47.2 [21.4,336]	81.9 [26.1,194.7]	0.49

**Surgery**	0.30

No	6 (37.5)	3 (27.3)	3 (60.0)

Yes	10 (62.5)	8 (72.7)	2 (40.0)

**EBRT + brachy^b^**	0.25

No	4 (25.0)	4 (36.4)	0 (0.0)

Yes	12 (75.0)	7 (63.6)	5 (100.0)

**Total Dose (Gy)**	69 [60,84]	60 [60,78]	81 [60,84]	0.07

**Chemotherapy**	0.31

No	7 (43.8)	6 (54.6)	1 (20.0)

Yes	9 (56.2)	5 45.5)	4 (80.0)

_______________________________________________________________________________________________________

**^a^**Students t-test, Wilcoxon test, or Fisher’s Exact test were utilized for normally distributed variables, non-normal variables

and categorical variables respectively. p<0.05 was considered statistically significant.

**^b^**External beam radiotherapy (EBRT) and brachytherapy (brachy)
