# Supplementary material for: Serum, cell-free, HPV-human DNA junction detection and HPV typing for predicting and monitoring cervical cancer recurrence
Source: eLife. 2026 Sep 9;14:RP105741. doi: 10.7554/eLife.105741 (PMC13557711; doi:10.7554/eLife.105741)
Supplement: Peer review history

# Editor report: eLife Assessment

**DOI:** 10.7554/eLife.105741.2.sa0

**Authors:** Lynne-Marie Postovit

**Affiliations:** Queens University Canada

**Keywords:** Incomplete, Valuable

This is a **valuable** study that suggests that HPV-human DNA junctions can be identified from cfDNA in women with cervical cancer and that detection of these junctions is indicative of recurrence. The evidence supporting the conclusions is **incomplete**, in part because the numbers of reads identifying breakpoints in tumor samples or in circulating cell-free serum samples are not provided. More quantitative analysis will be required to confirm that the breakpoints represented in cell-free DNA can be used as a surrogate to monitor the recurrence of cervical cancer cells, and additional patient studies would also be needed to strengthen the study. This work will be of interest to those who study and treat cervical cancer as well as other HPV-related malignancies.

# Referee report: Reviewer #1 (Public review)

**DOI:** 10.7554/eLife.105741.2.sa1

Van Arsdale and colleagues evaluated whether human-HPV DNA junctions could be detected in serum, cell-free DNA from 16 patients with cervical cancer by hybrid capture and Illumina sequencing. Junctions were identified in seven patients, and these junctions were concordant with junctions identified in tumor DNA except for one patient, suggesting that, in most cases, the cfDNA is originating from a clone of the primary tumor. Junction detection at 6 months was found to be statistically significant prognostic for recurrence. The study further validates that type-specific E7 DNA, which is essential for tumorigenesis, was detectable by PCR for most patient sera, but had no association with recurrence. Furthermore, the study provides additional evidence that tumors harboring non-alpha-9 clade HPVs had shorter recurrence-free survival and overall worse outcome from the study's patients, as well as reanalysis of TCGA data. However, these findings need to be more extensively discussed in the context of previous publications. One identified limitation of this approach is the detection of non-tumor HPVs, but this was only seen in one patient. The major shortcoming of this study is the limited number of patients that were evaluated, but for a retrospective study, this is a reasonable number of patients evaluated, and the findings are appropriately not overstated. The design, execution, and detailed analysis of the sequencing data are a major strength. This study provides important foundational evidence for further evaluating the clinical utility of HPV DNA detection from cfDNA and specifically assessing for integration junctions.

# Referee report: Reviewer #2 (Public review)

**DOI:** 10.7554/eLife.105741.2.sa2

Summary:

The authors set out to identify cell-free HPV breakpoint junctions and assess their utility in identifying cervical cancer recurrence as a surrogate, tumor-specific assay. They added unrelated findings about a potential relationship between various viral types and cancer recurrence frequencies, concluding that clade alpha 9 types recurred at a lower rate than did non-alpha 9 viral types.

Strengths:

The authors analyzed 16 cervical cancer samples and matched serum samples collected initially or upon clinical treatments. An association between virus types and cancer recurrence frequencies is a novel finding that will likely induce further insights into HPV pathogenic mechanisms.

Weaknesses:

The main claims of this manuscript are only partially supported by the data as presented, because the sequencing data are not quantified and were not analyzed in a statistically adequate way. First, only one or at most two breakpoints are presented per tumor (Table 1). This finding is discrepant from many extensive, published genomics studies of HPV-positive cancers, in which many unique breakpoints are found frequently in individual cancers, ranging from 1 or 2 up to more than 100. Second, no information is provided about likely correlations between genomic DNA copy number at rearranged loci and breakpoint-identifying sequencing read counts. Third, no direct comparison is presented between supporting read counts from cancer samples and read counts from circulating cell-free DNA samples. Fourth, many of the initial cancer samples harbored no insertional breakpoints, so no correlation with breakpoints in the serum samples would be possible. Fifth, no mention was made about tumor heterogeneity, where a given breakpoint may not be present in every cell of the tumor. Previous literature about the general topic of using cell-free DNA breakpoints as a surrogate for cancer cells is not cited adequately. Findings about potential correlations between various viral types and variable recurrence rates are not well-supported by the authors' own data, because of the limited sample numbers studied. This section of the paper is relatively unrelated to the main thrust, which is about breakpoint detection.

# Author comment: Author response

**DOI:** 10.7554/eLife.105741.2.sa3

**Authors:** Anne R Van Arsdale, Olga Meshcheryakova, Sonia Gallego, Elaine C Maggi, Bryan Harmon, Dennis Y Kuo, Koenraad Van Doorslaer, Mark H Einstein, Brian J Haas, Cristina Montagna, Jack Lenz

**Affiliations:** Albert Einstein College of Medicine Bronx United States; Albert Einstein College of Medicine Bronx United States; Albert Einstein College of Medicine Bronx United States; Albert Einstein College of Medicine Bronx United States; Montefiore Medical Center Bronx United States; Montefiore Medical Center Bronx United States; University of Arizona Tucson United States; Montefiore Medical Center Bronx United States; Broad Institute of MIT and Harvard Cambridge United States; Rutgers Cancer Institute of New Jersey New Brunswick United States; Albert Einstein College of Medicine New York United States

Both reviewers noted that some published studies question the association of HPV types with cervical cancer survival {PMIDs 36207323 and 33117670}, while others did not observe that {REFS 69-74 in Chakravarty}. We appreciate both reviewers pushing us to discuss and hypothesize (even speculate) on our finding that HPV types not in phylogenetic clade α9 types (including HPV18) had more recurrences than α9 types (including HPV16). The most likely explanation is that we analyzed 225 HPV types not just the most prevalent types. Specifically, each of the 5 recurrences in our pilot study had different HPV types (α7’s: 18, 39, 45, 70 & α5: 69). Similarly, on re-examination of the TCGA data set, we found that 80% of the 181 α9 samples had HPV16, while 52.5% of the non-α9 samples had HPV18, consistent with a broader variety of types in the latter. We note that PMID: 36207323 did assess a broad number of HPV types, but these were classified into three non-cladistic categories, HPV16, HPV18 and Other for comparison. More in line with the main point of that study, HPV18 was enriched, though not significantly, in the more pathogenic C2 group (which was defined by a deep analysis of specific genomic alterations). It can be speculated that perhaps α9 types are less proficient at effecting or interacting with some C2 characteristic(s). Overall, we suggest that these observations emphasize the importance of examining the full spectrum of HPV types including phylogenetic relationships in cervical cancers induced by these viruses.

**Reviewer #1:**

The detection of “non-tumor HPVs” was noted as a potential limitation. The highly multiplexed, HC+SEQ methodology that we use obviously detects many HPV types and thus can identify lesions with multiple HPV types as occurred in Patient 16 and in other HPV cancers. It is unclear what role multiple HPV types might play in tumorigenesis if any. Regardless of whether broad detection of HPV types proves to be a limitation or an advantage, it will be interesting. Our approach in this study focused on integration of HPV DNAs into human DNA, as this is a key event in cervical tumorigenesis. We believe that detection of clonally expanded cells with an integrated URR-E6-E7 DNA segment of any HPV type (whether high-risk, low-risk, or intermediate, or even perhaps non α-clade {PMID:40742260}) should be viewed with suspicion. For the small fraction of cervical cancers that contain only unintegrated HPV DNA, it will be interesting to see if these viral DNAs share any particular properties.

The reviewer asked for details of the HPV DNA capture probes used. All were from the proprietary Roche Nimblegen SeqCap EZ System. They encompassed all HPV types from HPV1 through HPV225.

The reviewer questioned why the data verifying the viral-human DNA junctions in primary tumor tissue by the orthogonal approach of PCR assays PCR assays were not shown. The data summary and the approach used for PCR are in Figure 1, Table 1 and Supplementary Table 1. Only the dozens of agarose gel photographs were not shown. PCR assays that addressed key issues comparing primary and metastatic sites and confirming HPV16 + HPV18 coinfection are shown in Figure 2 and Figures 4A & 4B, respectively.

**Reviewer #2:**

The reviewer raised general issues about data quantification and statistical adequacy. Regarding data quantification, we used a strict, conservative guideline of a 10 read minimum per junction in the DNA from tumor samples. This was based on the sequence analysis pipeline design and on our requirement that some clonal expansion of cells containing specific junctions must have occurred. Extensive complications to comparing quantified read counts in different studies are detailed below in the responses to specific comments. The statistical methods used were based on the dichotomous variable of detection versus no detection of integrated HPV DNA. For this study, we also used the orthogonal method of verifying every junction by PCR with one primer in viral DNA and the other in flanking human DNA followed by Sanger sequencing. The statistical methods used were entirely appropriate for this dichotomous variable and time to event analyses. Nonetheless, we concur that quantification of HPV DNA integration would be an interesting variable to consider once carefully controlled methodologies are applied considering the issues detailed below.

Regarding the first point about variability in HPV-human junction number in different studies: The number of HPV DNA genome and junction read counts obtained from a sample are subject to numerous technical and biological variables. Extensive caution should be applied when comparing quantitative results among different studies, and this particularly includes the number HPV-human DNA junctions detected. Among the factors that can be involved among different studies are the following: (1) inadequate deduplication of sequence reads; (2) “barcode-hopping” or “bleed-through” from one sample to another and thus cross-contamination of one sample with another during multiplexed short-read sequencing; (3) variation in the fraction of cells that are tumor cells in the post-clinical analysis sample of tissue obtained; (4) artifactual ligation of HPV and human DNA segments occurring at the adaptor ligation step of short-read sequencing; (5) variability in the mismatch settings of computational sequence aligners used; (6) perhaps most importantly, the level of genomic instability of each particular integration locus; and (7) subclonal variation in proliferation or survival of cells containing specific junctions within a lesion. The reviewer correctly implied that our requirement for a minimum of 10 sequence reads at each junction excludes low level, subclonal variants. Nonetheless, one tumor did have two integrations (Table 1). More importantly, we emphasize that all five tumor-recurrences at distant metastatic sites in our study had the exact same integration event as the primary tumor (determined at single nucleotide resolution at both ends). We judge this to be compelling evidence that the approach we use correctly identifies the key integration event underlying each cancer.

Regarding the second point about ratios between genomic DNA copy numbers and junction read counts: Both human genome and HPV genome copy numbers deserve mention in regard to this issue. HPV HC+SEQ highly enriches for viral DNA, with the advantage gained of high read depth for viral sequences, but with human DNA largely excluded (except for the junction reads). Thus, ratios of junctions to the rest of the human genome cannot be assessed as they can be with whole genome sequencing methodologies. While HPV genome read depth can be ascertained with HC+SEQ reads (as in Figure 1C, 1D, 1E), and the reviewer’s suggestion raises the possibility of using junction to viral read ratios to normalize data to compare different integration loci and even perhaps different studies, there are nonetheless additional, biomedically relevant complications. HPV DNA segments are sometimes often present as tandem units with or without human DNA segments in tumors (Figure 1E shows the former), and this affects the ratio of junctions to viral genomes. Thus, using the suggested ratios would require additional normalization for tandem copy numbers, and thus, it would be difficult to use them in a manner analogous to gene-specific read counts per million total read ratios in RNA-seq.

Regarding the third point about comparing read counts from primary tumor tissue with those from cfDNA: Ours was a retrospective study using archived samples that were available, and the HPV genome coverage obtained by HC+SEQ using cfDNA varied (Table 1). Assessment of viral DNA genome and human junction reads in a quantitatively reliable manner by HC+SEQ will require application of precise collection, storage, and processing of cfDNA samples. Nonetheless, the results presented in this study, while variable among the different samples, were entirely sufficient to test the dichotomous variable analyzed. We note that this included orthogonal, PCR verification of junctions, based on the straightforward, abundant identification of the junctions by HC+SEQ in the primary tumor samples. We emphasize that examination of HPV DNA integration directly interrogates a key, likely causal event in HPV cervical tumorigenesis.

Regarding the fourth point about many of the initial cancer samples harboring no junction breakpoints: 100% of the 16 initial, cervical, primary tumor tissue samples harbored an integration (one sample had two). The reviewer is correct that many of the initial cfDNA samples lacked HPV DNA integration as assessed by HC+SEQ and by PCR based on the junctions detected in the primary tumor tissue. We interpret this to mean that these cancers were not spilling genomic DNA containing the integrated HPV DNA into serum at sufficient levels to be detected, and judge this to be due to underlying, unidentified, biomedically-relevant effects.

Regarding the fifth point about HPV-human DNA junctions being used as a measure of tumor heterogeneity and subclonal variation: We concur with the reviewer that this is an interesting, important issue. We noted it in the response to the “first” point (numbers 6 and 7) above. Again, one of the samples had two integrations, and this patient did not suffer a recurrence (Table 1, Figure 1). Based on our ongoing experience, to take findings of junction subclonality beyond just detection of multiple integration junctions, we believe that development of in situ, single cell approaches are necessary to reveal the full meaningful picture of subclonality.

Beyond these quantitative issues that we raise in response to Reviewer #2’s comments, the Reviewers’ comments point at important, incompletely understood aspects about HPV tumorigenesis. Our finding of the identical viral DNA insertions in primary tumors and metastases point to a central, constant role for these structures in viral tumorigenesis. Nonetheless, the issues raised point to key questions concerning subclonality, detailed structures and quantification of HPV and human tandem DNA units, intrachromosomal DNA vs. ecDNA, genomic instability of integrated HPV DNA loci, and cell-to-cell variation, and what roles these might play in tumorigenesis.

Regarding the point about cell-free DNA breakpoints, we note the field of circulating tumor DNA fragmentomics that examines the sequences and a host of structural properties of circulating DNAs derived from tumors including specific, short sequences at the ends (breakpoints) of DNA fragments circulating in blood. These are of emerging significance as biomarkers for cancer {PMIDs:40038442 and 41043439}. We note that cell free DNA breakpoints are not synonymous with DNA junctions. We stress again that the main point of our manuscript was to investigate HPV-human DNA junctions in cfDNA, as this directly addresses a likely causal mechanism underlying HPV cervical tumorigenesis. Additional, future studies would be required to assess the effectiveness of our targeted, individualized approach relative to other aspects of fragmentomics in cervical cancer.

In summary, we restate one of the reviewers’ points. “This study provides important foundational evidence for further evaluating the clinical utility of HPV DNA detection from cfDNA and specifically assessing for integration junctions.” Both reviewers raised thoughtful points about DNA integration and HPV tumorigenesis, and prospective studies are required to refine and evaluate clinical utility of the new findings presented here.
